# Methylphenidate, but not citalopram, decreases impulsive choice in rats performing a temporal discounting task

**DOI:** 10.3389/fpsyt.2024.1385502

**Published:** 2024-05-08

**Authors:** Miranda F. Koloski, Alyssa Terry, Noelle Lee, Dhakshin S. Ramanathan

**Affiliations:** ^1^ Mental Health, VA San Diego Medical Center, San Diego, CA, United States; ^2^ Center of Excellence for Stress and Mental Health, VA San Diego Medical Center, San Diego, CA, United States; ^3^ Department of Psychiatry, University of California-San Diego, San Diego, CA, United States

**Keywords:** citalopram, impulsivity, methylphenidate, nucleus accumbens, temporal discounting

## Abstract

**Introduction:**

Drugs targeting monoamine systems remain the most common treatment for disorders with impulse control impairments. There is a body of literature suggesting that drugs affecting serotonin reuptake and dopamine reuptake can modulate distinct aspects of impulsivity – though such tests are often performed using distinct behavioral tasks prohibiting easy comparisons.

**Methods:**

Here, we directly compare pharmacologic agents that affect dopamine (methylphenidate) vs serotonin (citalopram) manipulations on choice impulsivity in a temporal discounting task where rats could choose between a small, immediate reward or a large reward delayed at either 2 or 10s. In control conditions, rats preferred the large reward at a small (2s) delay and discounted the large reward at a long (10s) delay.

**Results:**

Methylphenidate, a dopamine transport inhibitor that blocks reuptake of dopamine, dose-dependently increased large reward preference in the long delay (10s) block. Citalopram, a selective serotonin reuptake inhibitor, had no effect on temporal discounting behavior. Impulsive behavior on the temporal discounting task was at least partially mediated by the nucleus accumbens shell. Bilateral lesions to the nucleus accumbens shell reduced choice impulsivity during the long delay (10s) block. Following lesions, methylphenidate did not impact impulsivity.

**Discussion:**

Our results suggest that striatal dopaminergic systems modulate choice impulsivity via actions within the nucleus accumbens shell, whereas serotonin systems may regulate different aspects of behavioral inhibition/impulsivity.

## 1 Introduction

Impulsivity describes choosing immediate actions without considering future consequences and is observed in substance abuse, pathological gambling, attention disorders, schizophrenia, bipolar disorder and can result from frontal brain injury (1–9). Accordingly, there is a considerable body of literature on the neuropharmacological basis of impulsivity. Monoaminergic drugs are still considered the first line of treatment for many neuropsychiatric disorders for which impulsivity is a primary symptom.

Impulsivity has been categorized into two behavioral domains: impulsive action (the inability to inhibit an inappropriate response) and impulsive choice (choosing immediate rewards over more beneficial delayed rewards) (2, 3, 6, 10, 11). Delay discounting tasks are primarily used to measure impulsive choice by examining how decisions are made based on trade-offs between reward magnitude and temporal delay (12). Humans and animals both prefer actions leading to an immediate reward over actions where reward is delayed and discount in a hyperbolic fashion (2, 4, 9, 13–15). Generally, subjects select either a small reward delivered immediately, or a larger reward delivered after a temporal delay (2, 4, 9, 13–15). Impulsive choice in these tasks describes the inability to wait for a larger reward (2, 8, 9, 13, 14, 16). Preclinical neuropharmacological research has proven valuable to uncover how drugs interact with neural networks to change behavioral outcomes (17).

Several lines of research suggest that the ventral striatum and its cortical afferents regulate impulsive choice (7, 8, 18–25). Greater impulsivity is associated with more activity in ventral striatum (nucleus accumbens), and improved behavioral control is linked with top-down regulation of ventral striatum via medial prefrontal and orbitofrontal cortex is (25, 26). Delay discounting specifically recruits three brain networks: the reward network (ventral striatum, orbitofrontal cortex, medial prefrontal cortex), cognitive control network (anterior cingulate, dorsolateral prefrontal cortex) and a prospection network (hippocampus and amygdala) that change their within and between network connectivity in relation to discounting behavior (27, 28). Evidence from both animal studies and neuroimaging data from humans suggest the cortico-striatal network, and ventral striatum in particular, could be a potential therapeutic target for reward-guided impulsivity.

Cortico-striatal structures are regulated by dopaminergic and serotonergic systems which affect impulsive choice (1, 7, 15, 18, 29–31). Mesolimbic dopamine, originating from the ventral tegmental area terminating in ventral striatum, regulates behavioral activation, motivation, and reinforcing properties (12, 32, 33). Dopamine (and by proxy drugs that increase dopamine) generally reduce choice impulsivity on temporal discounting tasks (12, 34). Serotonin regulates mood, decision-making, learning, memory, sleep, locomotion, and social behaviors and presumably shape interactions between amygdala, hippocampus, and prefrontal cortex which contain a high number of serotonin receptors (35, 36). Premature, impulsive responses are associated with low serotonin levels (12, 36).

Monoaminergic drugs remain the first-line treatment for impulsivity and attention disorders. In humans, stimulants (like methylphenidate) are known to reduce impulsive behavior in attention-deficit disorders (37–43). Methylphenidate binds to dopamine and norepinephrine transporters to prevent reuptake, thus increasing neurotransmitter availability in cortical and subcortical structures including nucleus accumbens (44–47). Prior work has shown that increasing dopamine generally reduces choice impulsivity on temporal discounting tasks (4, 30, 48, 49) whereas dopamine antagonism can induce impulsive choice (4, 38, 50). Citalopram, a selective serotonin reuptake inhibitor (SSRI) is commonly used as an antidepressant and to treat behavioral control disorders, like substance abuse and obsessive-compulsive disorder (5, 51, 52). SSRIs (that increase serotonin), have also been shown to decrease action impulsivity (39, 53, 54). Serotonin levels are inversely related to impulsive actions, as measured on 5-choice serial reaction time task and go/no-go tasks (2). The relationship between serotonin and impulsive choice is not as clear. Lisdexamefetamine, a stimulant, increases effort output in rats significantly more than citalopram, suggesting that effort-based choice may be differently regulated by monoaminergic drugs (55).

In this study, we first test the dose-dependent effects of both methylphenidate and citalopram on choice impulsivity in a temporal discounting task. Clinically, both methylphenidate and citalopram are used to treat impulsivity and attention disorders. Based on previous literature, we predict that striatal activity is important for the regulation of choice impulsivity in temporal discounting tasks. If dopaminergic regulation in the ventral striatum is a critical for reward-guided choice, then we predict that increasing dopaminergic tone with methylphenidate, will decrease impulsivity, whereas citalopram will not affect impulsivity (12). To further test the association between ventral striatum and impulsivity, we lesion the nucleus accumbens and predict that the depletion of dopamine in this area will drive a decrease in choice impulsivity.

## 2 Materials and methods

### 2.1 Ethics statement

This research was conducted in strict compliance with the Guide for the Care and Use of Laboratory Animals of the National Institutes of Health. The following protocol was approved by the San Diego CA Medical Central Institutional Animal Care and Use Committee (IACUC; Protocol Number A17-014 and A21-012).

### 2.2 Animals

The subjects were 12 male Long-Evans rats obtained from Charles River Laboratories at approximately one month old (150g). Rats began training/habituation two weeks after arrival. Rats were housed in pairs upon arrival and then were single housed in standard cages (10 x 10.75 x19.5 inches) at the start of Experiment 1. Rats had free access to food and were kept on a 12 h light/dark cycle (6 AM/6PM). Behavioral testing occurred during the light cycle. During training, animals underwent water-scheduling, a form of water restriction where they receive free access to water for 2 hours/day to maintain motivation for water reward during operant behavior. Rats were weighed weekly to ensure that water-scheduling did not lead to reduced food intake/dehydration. Free access to water was provided on days with no behavioral training. The subjects were 4 months old when the study began.

### 2.3 Behavioral apparatus

Our custom operant chambers consist of 5 nose-ports each with an LED and IR sensor and were connected to a peristaltic stepper motor to pump water to nose-ports via clear tubing. Each chamber was fitted with two auditory tone generators and a house light. The apparatus is run using Raspberry PI module with MATLAB/Simulink software. The chamber design and programming has been described previously (56).

### 2.4 Behavioral training

#### 2.4.1 Pre-training

Animals began on a pre-training program to shape basic operant responses and habituate to the chamber. First, rats learned that a noseport with an LED “on” signaled an available response port and that a response in the available port would trigger an immediate water reward (500ms after response). Animals advanced to the next stage of training when they performed 100 trials in 60 minutes for three consecutive days. Next, rats trained on a version of the temporal discounting task where they could choose between two response ports that both would deliver water immediately (500ms after response). One choice led to a large reward (30µL) and the other to a small reward (10µL) delivered at a rate of 10µL/1s. Once rats showed a clear preference for the large reward choice (≥70% large reward choices/session) and consistently performed 100 trials, they advanced to the final temporal discounting task. Habituation and pre-training took 12 weeks to complete.

#### 2.4.2 Temporal discounting task

**Figure 1:**
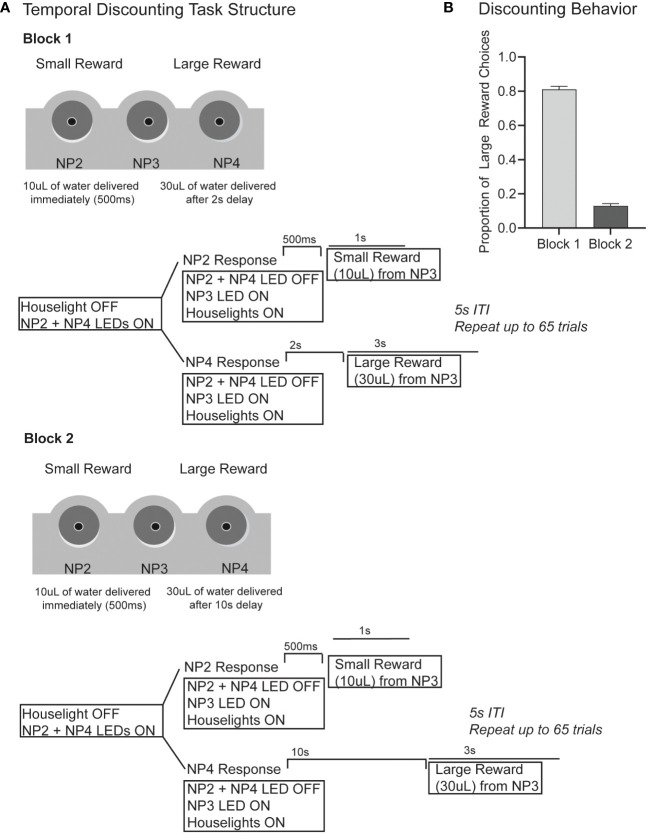
**(A)** The task structure for our version of the temporal discounting task is shown. Briefly, subjects must choose between a small reward (10µL) delivered immediately (500ms after response) and a large reward (30µL) delivered after a delay. In block 1 (first 65 trials) the large reward delay is 2s following response. In block 2 (>65 trials) the large reward delay is 10s). The schematics show the outline for a single trial with events including houselights, noseport (NP) LEDs, noseport responses and water delivery. **(B)** In block 1 when the large reward delay is short (2s), rats make 81% large reward responses. In block 2, when the large reward delay is long (10s), rats only make 13% large reward responses.

Generally, in a temporal discounting task subjects must choose between a low-value reward delivered immediately, or a higher-value reward delivered after a temporal delay. An impulsive choice describes the inability to wait for a larger reward (2, 8, 9, 13, 14, 16). In our version of the task, the subject must choose between a small reward (10µL) delivered immediately (500ms after response) or a large reward (30µL) delivered after a short (2s) or a long (10s) delay (**Figure 1**). Our version of the task is self-paced. A session began with 6 forced-choice trials, forcing the rat to sample both the small and large reward option without delays. The houselights started on, and LEDs in noseport 2 or 4 signaled the available choice response port. After either response, water was delivered from noseport 3 immediately (500ms after response). After the 6 forced-choice trials were complete, the houselights dimmed and LEDs in both noseport 2 and noseport 4 turned on, signaling a choice between the two ports. After a response was made, the choice noseport LEDs turn off, the houselights turn on, and noseport 3 LED turns on to signal water availability. If a small reward choice was made (noseport 2), then 10µLs of water was delivered (rate of 10µL/s) from noseport 3 immediately (500ms after response). If a large reward choice was made (noseport 4), then 30µLs of water was delivered (rate of 10µL/s) after a fixed delay. In block 1 (<65 trials), the fixed delay from response to reward was short (2s) (**Figure 1A**; top panel). In block 2 (≥65 trials), the fixed delay was long (10s) (**Figure 1A**; bottom panel). There were no cues to signal a change in delay length between blocks. A 5s inter-trial-interval was initiated after a response. Sessions lasted for 40 minutes and rats ran 5 days/week. On average, rats performed 200 trials per session.

The order of blocks (short and long delay) was not randomized. We chose this approach such that previous history of temporal delay would be consistent across sessions, and optimized for a self-paced task with a greater number of trials at each delay. This raises potential confounds: 1) rats are completing a fixed number of trials during the short delay (2s) block, and a variable number of trials in the long delay (10s) block, 2) motivational factors that arise across a session, and 3) the time frame of drug effects. Since the order of blocks was always consistent, motivational/satiety effects that exist have an equal chance of occurring on control/drug days, presumably allowing for a fair comparison between sessions. Rats generally maintain their large reward preferences and trial counts across saline control days. Previous research demonstrates that prior associates between rewards and delays do affect future discounting behavior (57), a problem we avoid by maintaining consistency between sessions. Lastly, IP injections of both methylphenidate and citalopram have effects that last at least 60 min (beyond the length of our sessions) (58, 59).

### 2.5 Experimental design

#### 2.5.1 Experiment 1

12 rats were used to test the dose-dependent effect of methylphenidate and citalopram on impulsive choice in a temporal discounting task. First, we tested intraperitoneal (IP) injections of methylphenidate at 5 doses (0.5, 1.0, 2.0, 5.0, 10.0 mg/kg) delivered in a pseudo latin-squares design. As a control, a saline injection was given the session prior to any drug session (i.e. Monday- saline; Tuesday-drug; Thursday- saline; Friday- drug). This pattern was repeated until each subject had experienced two repeated measures of each methylphenidate dose (20 sessions). After a 1-week washout period, the same experimental design was applied to test injections of citalopram at 5 doses (0.5, 1.0, 2.0, 5.0, 10.0mg/kg) delivered in a pseudo latin-squares design. Saline was given the session prior to any drug. This pattern repeated until each subject had experienced two repeated measures of each citalopram dose (at least 20 sessions).

All injections were given 20 minutes before the start of a behavioral session. Methylphenidate delivered via IP injections in the rat reaches peak concentration 15-30 min following injection and most studies find behavioral affects lasting 60-100 min after administration (58). Citalopram IP injections in a rat reaches peak concentration ~40 min following injection and has a half-life 1.5 hours (59).

#### 2.5.2 Experiment 2

Next, we tested whether the observed effects in Experiment 1 were dependent on an intact nucleus accumbens (NAc). The NAc, composed of the core and shell, is implicated in action and choice impulsivity (2, 7, 18, 31). Accumulating evidence from lesion, inactivation and stimulation studies show that core and shell divisions of NAc have different effects on both action and choice impulsivity (60, 61). After completing Experiment 1, 8 rats underwent a modified drug study following bilateral excitotoxic lesions to the nucleus accumbens shell (NAcSh). Based on our prior results we tested IP injections of methylphenidate at a low (1.0 mg/kg) and a high (10.0 mg/kg) dose delivered in a pseudo latin-squares design. As a control, a saline injection was given the session prior to any drug session. This pattern was repeated until each subject had two repeated measures of each drug dose (at least 8 sessions).

### 2.6 Drug administration

Each pharmacological test consisted of a two-day sequence; an intraperitoneal injection of vehicle (saline) on the first day and a drug injection on the subsequent day. Methylphenidate hydrochloride was purchased from Sigma-Aldrich (St. Louis, MO, USA) and citalopram hydrobromide was purchased from Torrent Pharmaceuticals (Basking Ridge, NJ). All drugs were mixed in 0.9% saline and injected at a final concentration of 1 ml/kg. All doses were calculated as salt and dissolved in 0.9% sterile saline (methylphenidate), or sterile saline and 1% dimethyl sulfoxide (DMSO)(citalopram). Experiment 1: Drugs (methylphenidate and citalopram) were delivered at 0.5, 1.0, 2.0, 5.0, 10mg/kg in a pseudo-latin squares design such that dose order was randomized for each subject. IP injections were given 20 minutes before the start of a behavioral session. Each subject received two repeated measures of each drug dose. Intermittent injections of saline and methylphenidate (dose order randomized for each subject) were followed by a 1-week washout period and then the study was repeated with intermittent injections of saline and citalopram (dose order randomized for each subject). The order of drug was not randomized (methylphenidate always came first for every subject). Experiment 2: Methylphenidate was delivered at 1.0 and 10.0 mg/kg in a pseudo-latin squares design. As a control, 1 mL/kg 0.9% sterile saline was administered. Drugs were administered through IP injections given 20 minutes before the start of a behavioral session. Each subject had two repeated sessions at each dose x drug. Methylphenidate injections were completed before moving to citalopram injections. Rats were given a 1-week washout period between drugs.

### 2.7 Nucleus accumbens lesion

#### 2.7.1 Surgery

Surgeries were aseptic and all instruments were autoclaved before start. The surgery was conducted in a stereotaxic frame under isoflurane anesthesia (SomnoSuite, Kent Scientific), with a body-temperature controlled heating mat (VWR). Before surgery, rats received a single dose of atropine (0.2 mg/kg) to diminish respiratory secretions, a single dose of dexamethasone (0.5 mg/kg) to decrease inflammation, and 0.5-1mL of 0.9% sodium chloride solution prior to surgery. The area of incision was cleaned with 70% ethanol and iodine solution. Bupivacaine (max 0.2 mL) was injected under the skin at the incision site as a local analgesic when the rat was anesthetized. A midline incision was made, the skin and periosteum were retracted. Using a microdrill (Stoelting), 4 holes (0.9mm diameter) were drilled through the skull at the bilateral lesion sites. On each hemisphere two holes were drilled at different AP locations to target NAcSh (Site 1: AP; +2.3, ML; ± 1.0mm, DV; 7.5mm. Site 2: AP; +1.3, ML; ± 1.0mm, DV; 7.5mm). Positions relative to bregma based on Paxinos & Watson coordinates/nomenclature (Paxinos & Watson, 2013). A 34-gauge beveled needle attached to a 10µL microsyringe (Hamilton, Reno, NV) was mounted on a syringe pump (WPI, Sarasota, FL) for intracerebral infusions. N-methyl-D-aspartate (NMDA) (Tocris Bioscience) was dissolved in 7.4 pH phosphate-buffered saline until a final concentration of 100mM (1g NMDA). NMDA solution was injected at a rate of 0.1 µL/min for a total of 0.1µL per site. The surgeon waited 1 minute for diffusion before removing the needle tip from the site. After infusion, holes were sealed with bone wax and the incision was sutured. Rats recovered from surgery on a heating pad. For post-op care rats received sulfamethoxazole and trimethoprim in their drinking water (60mg/kg per day for 8 days) to prevent infections and 1.0 mg/kg of meloxicam (S.C) for 3 d after surgery for pain management.

#### 2.7.2 Lesion verification

Histology was performed to confirm the lesion size and location. Rats were sacrificed under deep anesthesia (isoflurane) by transcardiac perfusion of physiological saline followed by 4% formalin. Brains were extracted and immersed in 4% formalin for 24 h and then stored in 30% sucrose until ready to be sectioned. Tissue was blocked in the flat skull position using a brain matrix (RWD Life Science Inc., CA, USA), sectioned into 3mm blocks and placed in cassettes for paraffin embedding. Cassettes were washed in phosphate-buffered saline and incubated in a series of ethanol dilutions to dehydrate tissue followed by xylene to clear tissue. Cassettes were moved into 60°C paraffin for 1 hour (x3) to infiltrate tissue. Each sample was placed in mold and embedded in paraffin. Once cooled, samples were removed from the mold and stored at room temperature until ready to section. Paraffin embedded blocks were sectioned at 5 µm with a microtome (Leica Biosystems) in the coronal plane. The section was floated on a warm water bath (42°C) until smooth and then mounted on a glass slide. Tissue dried in an upright position overnight at room temperature. Slides were then deparaffinized in a series of xylene and ethanol washes and stained with thionin. The lesion location and lesion volume were measured for each subject (N=8).

### 2.8 Statistical analysis

Our primary analyses examined how doses of methylphenidate and citalopram affected choice behavior on the temporal discounting task. Our primary measure was the proportion of large reward choices in each block per session. We examined trial count per session as a secondary dependent measure. We used linear mixed models opposed to repeated measures ANOVA (rmANOVA) which require two additional assumptions: 1) rmANOVA assumes compound symmetry (covariances are similar). Violations can lead to an increase in Type I errors (62, 63). 2) rmANOVA assumes the data is complete for each subject. Subjects without data must be excluded (62, 63). In contrast, linear mixed models do not depend on assumptions about variance-covariance matrix and can accommodate for missing data (both problems present in this data set). For each test described below, we made q-q plots to check for normal distribution and used Kolmogorov-Smirnov tests for normality. Additionally, we graphed the mean predicted values v. fixed factors separately for each subject to visualize independent slopes and intercepts (validation to include subject as a random factor in the model) (64).

Experiment 1: 12 male rats were tested on two drugs (methylphenidate and citalopram) at 5 doses with two repeated measures. We used separate models for methylphenidate and citalopram data to examine fixed factors [dose (0.0,0.5,1.0, 2.0, 5.0, 10.0 mg/kg), block (2s v. 10s large reward delay), session] and random factors [subject, dose order]. Session was a repeated measure. Experiment 2: 8 rats received nucleus accumbens shell lesions. We used a within-subjects pre-post research design to assess change in discounting as a response to accumbens lesion, without including a no intervention control group. The pre-post design allowed us to compare, for every subject, discounting behavioral and effects of methylphenidate across multiple time points (before and after lesion) while limiting the number of drugs exposures, but provides limited control over confounding variables without a separate control group (65). We used linear mixed models to compare behavioral performance pre- and post-lesion. First, we used a linear mixed model for saline only days to examine the fixed factors [lesion (pre v. post), block (2s v. 10s large reward delay), session] and random factors [subject, dose order]. Session was a repeated measure. Next, we ran a linear mixed model to include methylphenidate. Fixed factors [lesion (pre v. post), block (2 v. 10s large reward delay), doses (0.0, 1.0, 10.0 mg/kg), session] and random factors [subject, dose order], with session as a repeated measure.

To determine the best fit of each model, we measured the Akaike information criteria (AIC) and Bayesian information criterion (BIC) of four commonly used covariance models (compound symmetry, scaled identity, AR (1), and unstructured) (63, 64). The covariance models are different in how they treat correlated errors of repeated measures across time. Compound symmetry assumes homogenous variance, unstructured covariance specifies no patterns in covariance, autoregressive species that covariances are not equal but systematically decrease across repeated measures, and scaled identity assumes repeated measures are independent with equal variance (64). The scaled identity model provided the lowest AIC and BIC scores (63, 64). A p value less than 0.05 was considered significant. Significant results were followed with Bonferroni-corrected *post-hoc* comparisons. Behavior on the temporal discounting task was analyzed in IBM SPSS Statistics v.28 (New York, USA) and visualized in Graph Pad Prism v.9.

## 3 Results

### 3.1 Experiment 1

First, we wanted to investigate how choice impulsivity on a temporal discounting task was influenced by methylphenidate or citalopram to tease apart the unique influence of dopamine and serotonin respectively. In our version of the temporal discounting tasks, rats could choose between a small reward (10µL) delivered immediately (500ms after response) or a large reward (30µL) delivered after a delay. In block 1 (≤65 trials) the large reward was delivered 2s after response and in block 2 (>65 trials) reward was delivered 10s after response (**Figure 1A**). On saline control days, rats performed an average of 186 (+/- 33) trials, choosing the large reward 81% of trials in block 1 (2s large reward delay) and only 13% in block 2 (10s large reward delay), indicating that rats were discounting the large value reward based on the temporal delay (**Figure 1B**).

**Figure 2:**
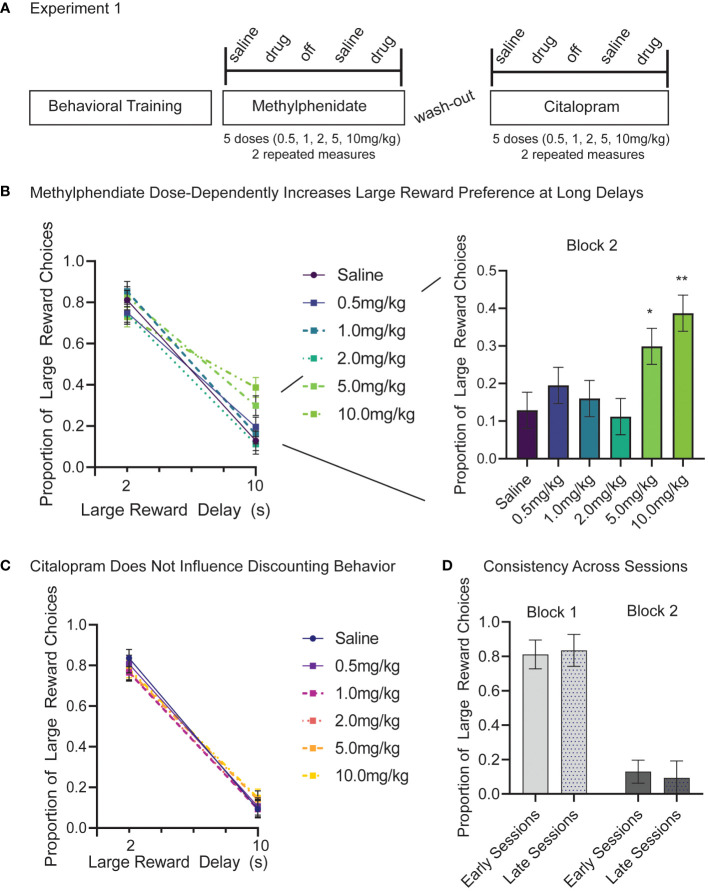
**(A)** Experiment 1 timeline and design. 12 male Long-Evans rats start Experiment 1 after 12 weeks of behavioral training and habituation. First, we tested intraperitoneal (IP) injections of methylphenidate at 5 doses (0.5, 1.0, 2.0, 5.0, 10.0 mg/kg). As a control, saline injections were given the session prior to a drug session. After a one-week wash-out period the study design was repeated using citalopram injections. Each subject completed two repeated measures at each drug and dose. **(B)** Methylphenidate dose-dependently increased the proportion of large reward choices but only in the long delay (10s) block (*p<.001*). There was no difference in large reward choices between saline and methylphenidate in the short delay (2s) block (*p=.191*). *Post-hoc* analyses reveal that, compared to the saline control, 5.0 mg/kg (*p=.017*) and 10.0 mg/kg (*p=.004*) of methylphenidate significantly increased the proportion of large reward choices in block 2. **(C)** Citalopram had no effects on proportion of large reward choice at any dose (*p=.908*) and there was no interaction between dose and block (*p=.751*). **(D)** Preference for large reward on block 1 (light) and block 2 (dark) did not change in control days from early sessions (methylphenidate) and late sessions (citalopram). Graphs show Mean and SEM. * *p<.05*, ** *p<.01*.

In Experiment 1, there were alternating days of saline and drug injections measured at 5 different doses (0.5, 1.0, 2.0, 5.0, 10.0 mg/kg) all repeated twice. The study started with methylphenidate injections and after a 1-week washout period was replicated with citalopram (**Figure 2A**). Data included is from 12 male rats across 288 sessions. We used separate linear models for methylphenidate and citalopram data. First, for methylphenidate days, we examined fixed factors of dose (6), block (2), and session, and random factors of subject and dose order. Session was treated as a repeated measure. This model showed significant changes in discounting behavior with methylphenidate injections. As observed on the saline control days, there was a main effect of block (reduced preference for large reward at a long delay), (F_(1,253.00)_=524.91, *p<.001*), but there was also a main effect of methylphenidate dose (F (5,253)=3.04, *p=.011*), and a significant interaction of dose and block (F_(5,253.00)_=4.56, *p<.001*). The *post-hoc* Bonferroni corrected tests show that methylphenidate had no effect on block 1 behavior when the delay was short (2s) (*p=.191*) but did dose-dependently increase the proportion of large reward choices in block 2 when the delay was long (10s) (*p<.001*) (**Figure 2B**). Compared to the saline control, 5.0mg/kg (*p=.017*) and 10.0mg/kg (*p=.004*) of methylphenidate significantly increased the number of large reward choices during block 2 (**Figure 2B**; right panel). High doses of methylphenidate made the rats more willing to wait 10s for a large reward. There was also a significant effect of methylphenidate on number of trials completed within a session (F (5,121)=10.39, *p<.001*) (**Supplementary Figure 1A**). The highest dose of methylphenidate (10.0 mg/kg) significantly reduced trials compared to the saline days (p*<.001*), which is related to choosing the long delay more frequently (cannot complete as many trials in the given timeframe).

In the same animals (N=12), we ran a second linear mixed model to examine the effects of citalopram on discounting behavior. The model included fixed factors of dose (6), block (2), and session, and random factors of subject and dose order. Session was treated as a repeated measure. The model still showed a significant main effect of block (F_(1,253.00)_=829.73, *p<.001*), but no other effects were significant. As seen previously, rats chose the larger reward more when the delay is short (block 1), and citalopram had no effects on large reward choice at any dose (*p=.908*) or interaction between dose and block (*p=.751*) (**Figure 2C**). Likewise, there is no difference in the number of trials completed per session based on citalopram injections (*p=.102*) (**Supplementary Figure 1B**).

The effect of session did not contribute significant variance to the models (*p=.273* methylphenidate; *p=.469* citalopram) nor did choice behavior change following the washout period (block 1 t (92) = 0.97, *p=.558*; block 2 t (92) = 1.46, *p=.275*) (**Figure 2D**). In saline control days before the washout period (early sessions) proportion of large reward trials was 0.81 (+/- 0.08) in the short delay block and only 0.13 (+/-0.07) in the long delay block. In saline control days following the washout period (late sessions) the proportion of large reward trials was 0.84 (+/- 0.09) in short delay blocks and 0.09 (+/-0.10) in long delay blocks (**Figure 2D**). Animals completed more trials in late sessions following the washout period (217+/-58) compared to earlier sessions (186 +/- 33) (t (46) = 2.30, *p=.026*) (**Supplementary Figure 1C**).

### 3.2 Experiment 2

Next, in the same cohort of rats, we repeated the methylphenidate study following excitotoxic bilateral lesions of NAcSh. The core and shell are functionally and anatomically distinct subregions of ventral striatum with studies supporting separate roles for each in regulating reward-guided behavior (60, 61, 66–68). NAcSh is implicated in reward-value processing and appetitive behavior (66–68). Lesions to NAcSh decrease premature or impulsive responding on a forced choice instrumental learning task (69). Therefore, we hypothesized that NAcSh lesions would reduce impulsivity on the temporal discounting task (more large reward choices at long delays), similar to the effects of methylphenidate observed in Experiment 1.

**Figure 3:**
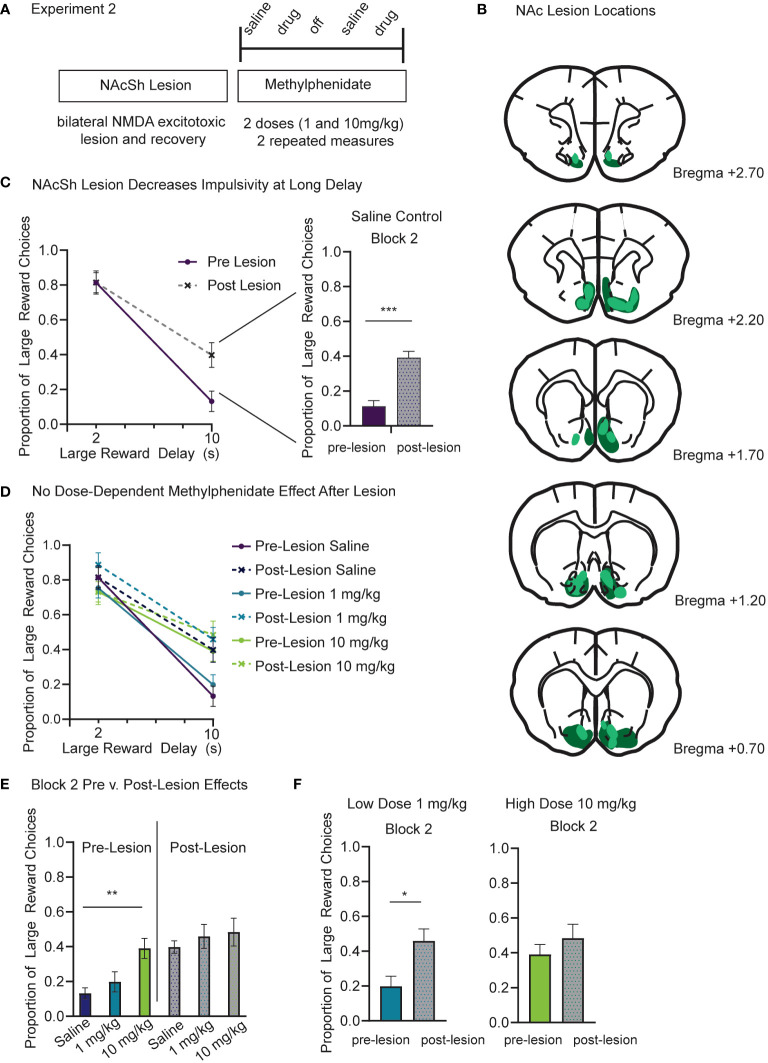
**(A)** Experiment 2 timeline and design. In the same cohort of rats, we repeated a modified drug study based on results from Experiment 1. First, rats received an excitotoxic NMDA lesion centered over bilateral NAcSh. Rats recovered for one week following surgery and then began the modified drug study. IP injections of methylphenidate were given at two doses (1.0 and 10.0 mg/kg). As a control, saline injections were given the session prior to a drug session. Each subject completed two repeated measures at each drug and dose. **(B)** Schematic showing the lesion sites at coronal sections from bregma AP +2.70 to +0.70. Dark green represents the largest lesion and light green represents the smallest lesion at each section as seen from the thionin stained tissue. **(C)** The NAc lesion increased the proportion of large reward choices in the long delay (10s) block (*p<.001*) and did not change choice behavior in the short delay (2s) block (*p=.203*). These results only include saline control days from pre- and post-lesion sessions. **(D)** The NAc lesion increases preference for large reward in the long delay (10s) block to a similar level as the 10mg/kg methylphenidate dose from Experiment 1. **(E)** Only the pre-lesion data (color) show a dose-dependent effect of methylphenidate (p=.005) in block 2. Post-lesion data (grey scale) show no difference between the saline, low and high methylphenidate doses. **(F)** Compared to pre-lesion data (color), the proportion of large reward choices during block 2 was greater following the lesion (grey scale) during 1mg/kg methylphenidate sessions (*p=.030*) but was no different in 10 mg/kg methylphenidate sessions (*p=.554*). Graphs show Mean and SEM. * *p<.05*, ** *p<.01*, *** *p<.001*.

One week after surgery rats began a modified version of Experiment 1. Based on our previous results showing a dose-dependent effect of methylphenidate and no effect of citalopram, we only tested a low (1.0mg/kg) and a high (10.0mg/kg) dose of methylphenidate on temporal discounting task performance in the lesioned animals (**Figure 3A**). The approximate lesion sites are shown which centered around NAcSh and extended from Bregma AP +2.75 to +0.50 (**Figure 3B**). Due to attrition, only 8 male rats completed Experiment 2. Post-lesion results are based on 96 sessions. The pre-lesion data used for statistical comparison is still based on 12 subjects.

First, we used a linear mixed model to compare each subject’s pre- and post-lesion data for saline days to determine the effects of accumbens lesion, before considering the additional effects of drug. The model included fixed factors of lesion (2), block (2), and session, and random factors of subject and dose order. Session was a repeated measure. The model found a significant difference between pre- and post-lesion data. There was still a main effect of block (F_(1,142.14)_=667.14, *p<.001*), such that preference for the large reward was reduced at long delays, but there was also a main effect of lesion (F_(1,149.36)_=38.64, *p<.001*), and an interaction of lesion and block (F_(1,142.14)_=36.91, *p<.001*). There was no difference in proportion of large reward choices in the short delay (2s) block after the NAcSh lesion (*p=.203*) (**Figure 3C**). However, lesioning the NAcSh increased the proportion of large reward choices during the long delay (10s) block (t (81)=6.84, *p<.001*) (**Figure 3C**; right panel). There was also a significant effect of lesion on number of trials completed (t (81)= 9.89, *p<.001*). After the accumbens lesion, trials were reduced by a mean difference of 85 +/-10, which is related to the increased preference for long delay choice (**Supplementary Figure 2A**).

Next, we ran a second linear mixed model to include effects of methylphenidate. This model included fixed factors of lesion (2), block (2), dose (3), and session, and random factors of subject and dose order. Session was treated as a repeated measure. If the effects of methylphenidate on choice impulsivity observed in Experiment 1 were dependent on NAc, then methylphenidate injections following the lesion would not change choice behavior compared to saline control days. Beyond the known effects of lesion (*p<.001*), block (*p<.001*), and their interaction (*p=.021*) as discovered with previous linear mixed models, there was also a significant interaction between methylphenidate dose and block (F_(2, 203.44)_=4.64, *p=.011*) that was again driven by the high dose methylphenidate (10mg/kg) increasing the proportion of large reward choices during the long delay (10s) block (**Figure 3D**). Only the pre-lesion data showed a dose-dependent methylphenidate effect on proportion of large reward choices during block 2 (F (2, 69)= 5.842, *p =.005*) (**Figure 3E**). The post-lesion data showed no difference between the saline, low dose and high dose methylphenidate groups (*p=.843*) (**Figure 3E**). Importantly there was no interaction between methylphenidate and lesion. Compared to pre-lesion data, the proportion of large reward choices during the long delay (10s) block was greater following lesion during 1 mg/kg methylphenidate injection sessions (t (39)=2.249, *p=.030*) (**Figure 3F**). However, there was no difference in the proportion of large reward choices during the long delay (10s) block during the 10mg/kg injection sessions (*p=.554*) (**Figure 3F**). Following the lesion, the proportion of large reward choices (.48 +/-.08 SEM) was similar to the effects of large dose methylphenidate before the lesion (0.39 +/-.06 SEM) (**Figure 3F**). There was a main effect of methylphenidate dose on trials (F_(2,107.15)_=19.46, *p<.001*) and a significant interaction between lesion and methylphenidate dose (F_(2,107.15)_=11.00, *p<.001*). Following the lesion there was a significant reduction in trials during saline (*p<.001*) (**Supplementary Figure 2A**) and low dose (1.0 mg/kg) (*p<.001*) (**Supplementary Figure 2B**) sessions, reflecting the increase of long delay trials after lesion. There was no significant difference in trial count for the high (10.0 mg/kg) methylphenidate injection (*p=.192*) (**Supplementary Figure 2C**).

## 4 Discussion

Monoamines regulate inhibitory control processes, but each neurotransmitter system likely influences a distinct behavioral domain (3, 7, 70). The targeted behaviors attributed to monoaminergic drugs is still largely unknown in part because the effects of different drugs have often been tested in different animals with different behavioral paradigms. Preclinical paradigms with high translational validity may offer the consistency needed to study processes under the umbrella of inhibitory control that contribute to maladaptive impulsivity (15).

Here, we use a temporal discounting task to study the contributions of dopaminergic and serotonergic systems on choice impulsivity. In Experiment 1 we tested dose-dependent effects of methylphenidate (dopaminergic) and citalopram (serotonergic) on a rat’s choice to wait (2 or 10s) for a large reward or receive a small reward immediately. In Experiment 2, we gave the same rats a NAcSh lesion and compared the effects of methylphenidate injections before and after the lesion. Prior work has shown that increasing dopamine generally reduces choice impulsivity on temporal discounting tasks (4, 30, 48, 49), but the effects of citalopram on temporal discounting tasks are not as consistent. Our results extend on these previous findings in four important ways: 1) Consistent with the above, methylphenidate, and not citalopram, decreased impulsive choice on the temporal discounting task. Methylphenidate injections increased the rats’ preference to wait 10s for a large reward (30µL) over a small reward (10µL) delivered immediately (500ms). 2) The effects of methylphenidate to reduce impulsivity were dose dependent. Only high doses (5.0 and 10.0 mg/kg) of methylphenidate increased the rats’ preference to wait for a large reward across a long delay period (10s). 3) Bilateral lesions to NAc also reduced impulsive choice. The dose-dependent effect of methylphenidate was not significant following a NAc lesion. The lesion reduced impulsivity to a similar level as high dose methylphenidate, and therefore methylphenidate did not have any additive effects following the lesion. 4) Modulations to the dopamine system (both methylphenidate injections and NAc lesions) only changed behavior during block 2 when the temporal delay was long (10s). Therefore, dopaminergic control may be uniquely important when reward-guided decisions involve weighing tradeoffs between magnitude and temporal delay.

In our study, citalopram had no effects on choice impulsivity. Although serotonin is known to be involved in behavioral control processes, it may not be mediating choice impulsivity when value-based decisions are regulated by temporal delay. Our results are consistent with prior studies showing that global 5-HT depletion does not affect delay discounting (71). Likewise, 5-HT depletion does not alter effort-based choice and citalopram cannot rescue motivational impairments (33, 55). A larger body of evidence suggests serotonergic agents affect action impulsivity (3, 54, 72–76). Citalopram specifically, reduces premature responding on the 5-choice serial reaction time task (3) and improves behavioral performance on a probabilistic reversal learning task (42). Our work is consistent with the larger idea that serotonin modulates action, but not choice impulsivity. More recent evidence shows that optogenetic stimulation of serotonin neurons in the dorsal raphe nucleus promotes waiting behavior (77), and firing rates of single dorsal raphe neurons are elevated while animals wait for delayed rewards (78), together suggesting that serotonin may specifically modulate the waiting component of inhibitory control. The effects of serotonin may also depend on receptor subtype. 5-HT_2A_ antagonism decreases premature responding (72), whereas 5-HT_2C_ antagonism enhances impulsive action (72, 73). Many serotonergic manipulations (depletion, selective lesions, receptor antagonists) are not divorced from dopamine systems (71), yet we, and others, find that important distinctions between the two systems exist.

Our results are in agreement with prior work showing that temporal discounting in both humans and rats is sensitive to dopaminergic manipulations. Striatal dopamine is increased following methylphenidate exposure (40, 41, 45, 47, 79–81), with this increase in dopamine likely subserving aspects of impulse control (47, 81), risky decision-making (82), regulation of reward (83, 84) and its effects on impulsivity (1, 30, 47, 85, 86). Low levels of dopamine receptors (D2/D3) in the NAc are associated with high impulsivity in outbred rodent models (87). Dopamine agonists decrease choice impulsivity whereby antagonists (specifically D2 receptors) increase choice impulsivity on temporal discounting tasks when delays change within (38, 50, 88) or between sessions (4). Although we attribute many of these effects to dopamine, methylphenidate also inhibits norepinephrine reuptake (44–46). Atomoxetine, a selective norepinephrine reuptake inhibitor, is often used to separate norepinephrine mechanisms from the combined effects of dopamine/norepinephrine seen with methylphenidate (37). On a delayed discounting task, atomoxetine dose-dependently decreased choice impulsivity (89). Future work must demonstrate whether the effects observed with methylphenidate on this task are linked with only dopamine or also occur as a consequence of norepinephrine reuptake inhibition. Similarly, dopamine is involved in arousal and attentional process that may affect temporal discounting performance. For example, accumbens dopamine depletions alter effort-based decision-making (32) and therefore the overlap between reward, motivational, and arousal effects of dopamine must be considered.

The NAc has two functionally and anatomically distinct subdivisions: the core and shell (18, 31, 60, 61, 66). Damage to the core, but not the shell, increases impulsive choice on temporal discounting tasks and alters effort-based motivation (61, 90). Likewise, stimulating the NAcC reduced premature responses on a rodent reaction time task, whereas shell stimulation induced premature responses (60). The nucleus accumbens core and shell are both innervated by dopamine, but only the shell receives norepinephrine (86), a distinction that could underscore the behavioral differences between the two subregions. This work largely suggests that increased activity within NAcSh is linked with impulsive behaviors whereas increased activity of the NAcC may be involved in behavioral inhibition. Consistent with this model, we found that NAcSh lesions decreased impulsive responding and made rats more willing to wait a longer delay for a larger reward. The lesion resulted in a behavioral effect which matched that achieved with the high-dose methylphenidate injection. After the NAcSh lesion, methylphenidate had no additive effect in reducing impulsivity, suggesting that the increased striatal dopamine that occurs with methylphenidate was acting via some form of inhibition of the accumbens shell (either directly or indirectly), and thus the “functional suppression” induced by methylphenidate was replicated by the lesion.

There are several caveats to this interpretation. First, because methylphenidate was administered systemically, we don’t have evidence that behavioral results observed are related to a direct effect of dopamine in the accumbens, but others have found that increased impulsivity following NAcSh stimulation was also associated with increased levels of dopamine and serotonin locally in the NAc, suggesting that ventral striatum does regulate dopaminergic levels (91). Second, lesions to the NAcSh may indirectly increase basal dopamine levels. The optimal level of dopamine to regulate impulsive decision-making likely follows an inverted U-shaped curve, as has been documented for other cognitive processes (44, 92–94), and thus too much dopamine (provided by methylphenidate) would not necessarily continue to improve behavior following the lesion. Third, reward-guided decision making relies on a large, distributed network, and thus lesions to a single brain area do not ascribe specificity as only the NAcSh being important for behavior. Indeed, it is possible that lesions across multiple parts of the distributed amygdala-cortico-striatal network involved in reward-based decision making could have similar behavioral effects and similar effects on methylphenidate. Delay discounting behavior depends on the functional integrity of cortical (orbitofrontal) and striatal (nucleus accumbens) regions (69, 90, 95). Although prefrontal cortex is important for reward-guided decision-making, inhibition involving delays to reinforcement are insensitive to medial prefrontal lesions (90). Thus, delay discounting relies on the interaction of multiple neural systems and cannot be determined by a single brain region (27, 28, 96).

Finally, there are methodological considerations. The excitotoxic lesions made here, do partially extend to the core, and do not ablate the whole shell division. Combined core and shell lesions also increase preference for larger delay rewards (97). Partial inactivation of the NAcC decrease delay discounting - opposite of what previous studies would predict based on complete lesions/inactivation (98). The NAc, and its associated dopamine terminals, left intact may modulate dopaminergic tone to positively influence choice impulsivity.

Our study adds to converging evidence from animal and human studies finding dopaminergic signaling within the nucleus accumbens and cortico-striatal network at large is a potential therapeutic target to treat symptoms of reward-related impulsivity. There are potential challenges when translating animal research to human populations including drug dosage and route of administration. IP injections of <2mg/kg methylphenidate produce plasma drug levels in rodents that fall within the clinical range of 8-40ng/mL (42). Here, we only show significant effects at doses >5mg/kg and thus the translational relevance of our dose-dependent effect is still not clear. Moreover, this study was only completed in male rats. Sex specific effects on temporal discounting is mixed and therefore what to predict is not clear (10, 99–101). Female rats show greater preference for small, immediate reward (impulsive choice), but this effect seems to be mediated by type of reward, delay length, and age/strain of rat (10, 100). Clinically, males exhibit higher rates of impulsive disorders (substance use and attention deficit disorders) (102, 103), providing motivation to start in a cohort of male rats, but the necessity to extend these findings to a female cohort is of top priority. Lastly, the interaction between environmental and biological factors cannot be ignored and may present differently in human and animal subjects. For instance, the interplay between stress and inflammation impacts brain connectivity of regions involved in decision-making (prefrontal cortex, amygdala) and may contribute to impulsivity disorders (104).

## 5 Conclusions

Our research adds to a growing body of literature showing discernable differences in the neural circuits underlying different forms of impulsivity and decision-making. This study highlights a dissociable neurochemical effect between methylphenidate and citalopram during choice impulsivity. Our data demonstrates a selective effect of methylphenidate and not citalopram to improve impulsive choice. A further investigation of the neurological mechanisms associated with these neurochemical effects will be critical for defining decision-making impairments that are central to a number of psychiatric disorders.

## Data availability statement

The raw data supporting the conclusions of this article will be made available by the authors, without undue reservation.

## Ethics statement

The animal study was approved by San Diego VA Medical Center Institutional Animal Care and Use Committee (IACUC; Protocol Number A17-014 and A21-012). The study was conducted in accordance with the local legislation and institutional requirements.

## Author contributions

MK: Conceptualization, Data curation, Formal analysis, Funding acquisition, Methodology, Project administration, Visualization, Writing – original draft, Writing – review & editing. AT: Data curation, Formal analysis, Investigation, Methodology, Project administration, Writing – original draft. NL: Data curation, Investigation, Methodology, Writing – original draft. DR: Conceptualization, Funding acquisition, Methodology, Supervision, Writing – review & editing.

## Supplementary material

Supplementary Figure 1 Trial number was analyzed as a secondary dependent measure in Experiment 1. **(A)** The 10 mg/kg dose of methylphenidate significant reduced trial number compared to the saline control (p<.001), which reflects the increase in long delay trials (cannot complete as many trials in the given timeframe). **(B)** There is no significant difference in the number of trials completed between saline control and any citalopram dose (p=.102). **(C)** Animals completed more trials in saline control days during late sessions (citalopram) compared to early sessions (methylphenidate) (p=.026). Graphs show Mean and SEM. * p<.05, ** p<.01, *** p<.001.

Supplementary Figure 2 Trial number was analyzed as a secondary dependent measure in Experiment 2. **(A)** After the NAc lesion (grey scale), there was a significant reduction in trials compared to pre-lesion (color) saline control days (p<.001). This difference reflects the increase in long delay trials (cannot complete as many trials in the given timeframe). **(B)** During 1 mg/kg methylphenidate injections, the number of trials was lower post-lesion (grey scale) compared to pre-lesion (color) sessions (p<.001). **(C)**. There was no difference in trial number in pre-lesion (color) or post-lesion (grey scale) sessions during 10 mg/kg methylphenidate injections. Graphs show Mean and SEM. * p<.05, ** p<.01, *** p<.001.
