# Supplementary figures and images for: Methylphenidate, but not citalopram, decreases impulsive choice in rats performing a temporal discounting task
Source: Front Psychiatry. 2024 May 8;15:1385502. doi: 10.3389/fpsyt.2024.1385502 (PMC11109432; doi:10.3389/fpsyt.2024.1385502)

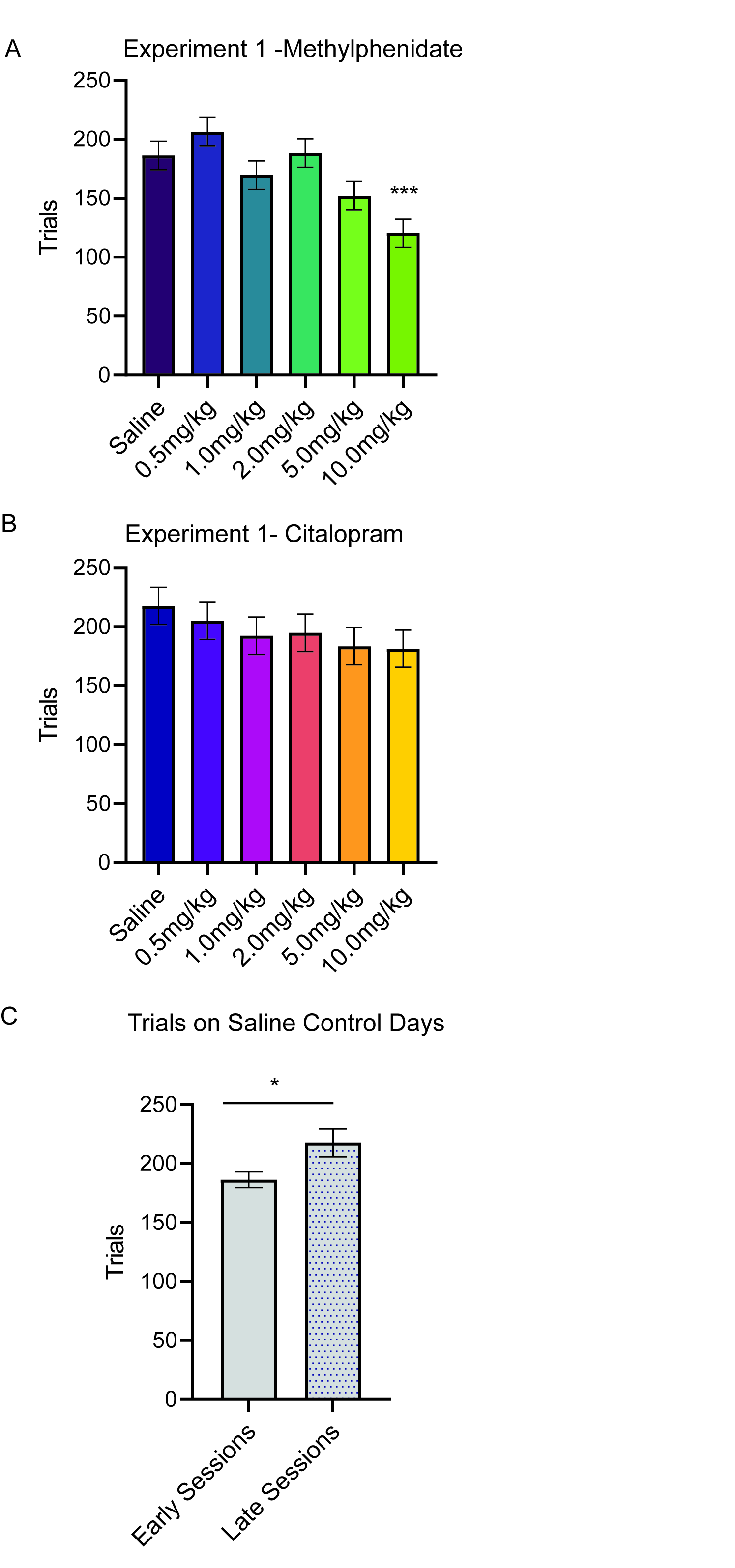

Supplement: Supplementary Figure 1 — Trial number was analyzed as a secondary dependent measure in Experiment 1. (A) The 10 mg/kg dose of methylphenidate significant reduced trial number compared to the saline control (p<.001), which reflects the increase in long delay trials (cannot complete as many trials in the given timeframe). (B) There is no significant difference in the number of trials completed between saline control and any citalopram dose (p=.102). (C) Animals completed more trials in saline control days during late sessions (citalopram) compared to early sessions (methylphenidate) (p=.026). Graphs show Mean and SEM. * p<.05, ** p<.01, *** p<.001. [file Image_1.tif]

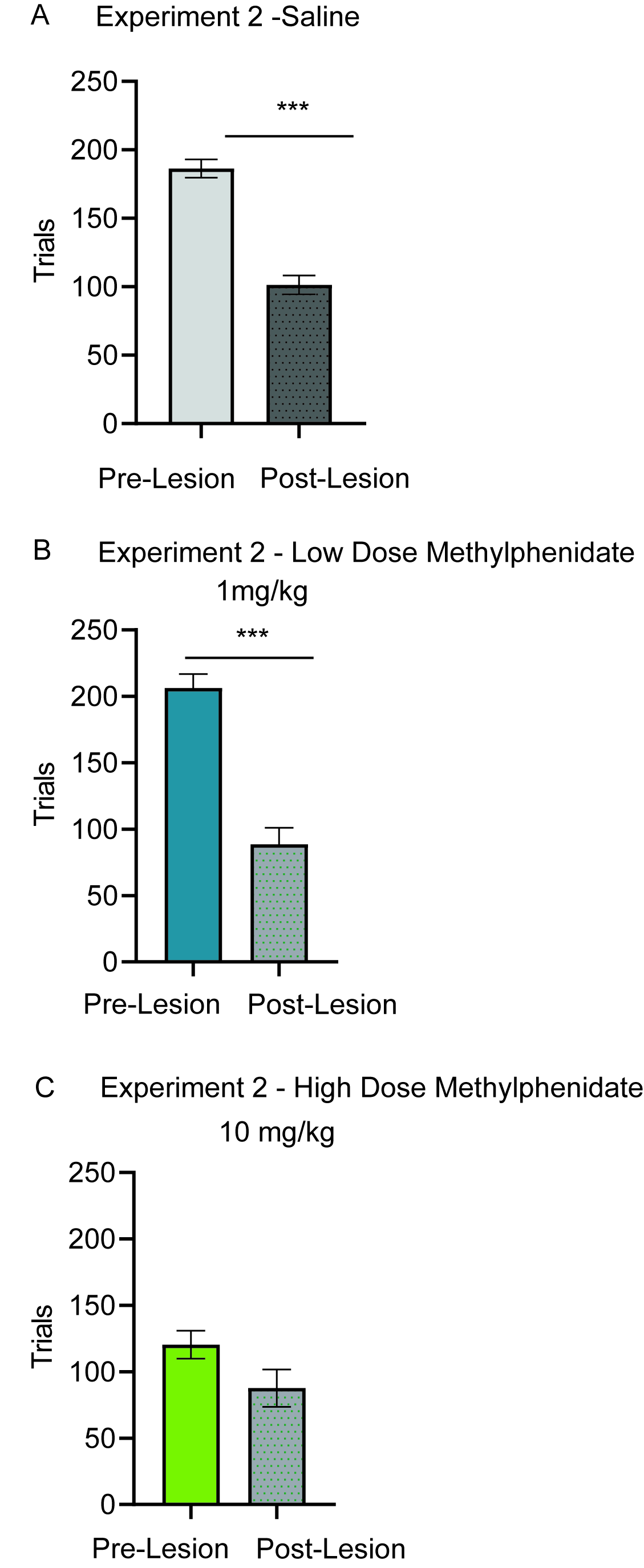

Supplement: Supplementary Figure 2 — Trial number was analyzed as a secondary dependent measure in Experiment 2. (A) After the NAc lesion (grey scale), there was a significant reduction in trials compared to pre-lesion (color) saline control days (p<.001). This difference reflects the increase in long delay trials (cannot complete as many trials in the given timeframe). (B) During 1 mg/kg methylphenidate injections, the number of trials was lower post-lesion (grey scale) compared to pre-lesion (color) sessions (p<.001). (C). There was no difference in trial number in pre-lesion (color) or post-lesion (grey scale) sessions during 10 mg/kg methylphenidate injections. Graphs show Mean and SEM. * p<.05, ** p<.01, *** p<.001. [file Image_2.tif]
